# Molecular Epidemiology of *Babesia vogeli* and *Hepatozoon canis* in Dogs from Urban and Peri-Urban Areas of Rio de Janeiro, Brazil

**DOI:** 10.3390/pathogens15040383

**Published:** 2026-04-02

**Authors:** Mariana Santos Ribeiro, João Pedro Siqueira Palmer, Laís Verdan Dib, Camila Souza Carvalho Class, Lucas Fernandes Lobão, Fabiana Batalha Knackfuss, Alynne da Silva Barbosa

**Affiliations:** 1 Departamento de Microbiologia e Parasitologia, Instituto Biomédico, Universidade Federal Fluminense, Rua Outeiro de São João Batista, s/n, Centro, Niterói 24020-141, RJ, Brazil; marianasr@id.uff.br (M.S.R.); joao_palmer@hotmail.com (J.P.S.P.); laisverdandib@gmail.com (L.V.D.); camilaclass@id.uff.br (C.S.C.C.); lucaslobao@id.uff.br (L.F.L.); 2 Parasitologia, Faculdade de Medicina de Campos, Alberto Torres Avenue, 217, Centro, Campos dos Goytacazes 28035-581, RJ, Brazil; 3 Zootecnia e Estatística, Universidade do Grande Rio, Rua Professor José de Souza Herdy, 1160, Jardim Vinte e Cinco de Agosto, Duque de Caxias 25071-202, RJ, Brazil; fbknackfuss@hotmail.com

**Keywords:** *Babesia vogeli*, *Hepatozoon canis*, tick-borne diseases, molecular epidemiology, domestic dogs

## Abstract

*Babesia vogeli* is considered endemic in urban settings of Brazil, whereas *Hepatozoon canis* remains insufficiently documented in several regions, including the metropolitan area of Rio de Janeiro. This study investigated the frequency, spatial distribution, and determinants of infection by piroplasmids and *Hepatozoon* spp. in dogs from distinct environments. A total of 372 blood samples from pet dogs were collected between June and October 2023 in Maricá (Area 1; *n* = 105) and in the Administrative Regions of Barra da Tijuca, Guaratiba, and Jacarepaguá (Area 2; *n* = 267). Molecular screening was performed using 18S rRNA gene-based PCR assays, followed by sequencing and phylogenetic inference. Conventional PCR was used for piroplasmids, while both conventional and nested PCR were applied for *Hepatozoon* detection. Overall, 30 dog samples (8.1%) tested positive. Piroplasmids were detected in 3.5% of dogs, with a higher occurrence in Area 1, whereas *Hepatozoon* spp. infection was identified in 5.4% of samples, with co-positivity with piroplasmids being rare. All piroplasmid sequences corresponded to *B. vogeli*, while *H. canis* was confirmed in thirteen dogs. The absence of owner-reported tick-borne disease history was the main factor associated with hemoparasite positivity. These findings provide the first molecular epidemiological evidence of *H. canis* circulation in different areas of Rio de Janeiro and highlight the need for integrated diagnostics, surveillance, and targeted actions to improve control.

## 1. Introduction

Canine piroplasmosis and hepatozoonosis are globally distributed tick-borne diseases that affect domestic dogs. Both are caused by hemoprotozoa of the phylum Apicomplexa transmitted by infected ixodid ticks [1,2]. Among the piroplasmids infecting dogs, *Babesia*, *Theileria*, and *Rangelia* are the most relevant taxa [3,4,5,6]. The species most frequently associated with canine piroplasmosis include *Babesia canis*, *B. vogeli*, *B. rossi*, and *B. gibsoni* [7]. In Latin America, *B. vogeli* predominates in domestic dogs, although sporadic occurrences of *B. gibsoni* have been reported [8]. Clinically, canine babesiosis may result in fever, hemolytic anemia, hemoglobinuria, and thrombocytopenia, among other abnormalities [1,9]. In dogs, *Babesia* spp. infection occurs when infected ixodid ticks inoculate sporozoites during blood feeding. The parasites invade erythrocytes, multiply by merogony, and release merozoites that perpetuate cycles of parasitemia. Some merozoites differentiate into gamonts, which are ingested by ticks, where sexual reproduction occurs in the midgut, leading to the formation of kinetes. These disseminate to the salivary glands and ovaries, enabling transstadial and transovarial transmission. Infective sporozoites are subsequently produced in the salivary glands and transmitted to a new vertebrate host during tick feeding, completing the life cycle [1].

Canine hepatozoonosis is caused by *Hepatozoon* spp. The transmission to dogs occurs through ingestion of a tick harboring sporulated oocysts [10]. Following release in the gastrointestinal tract, sporozoites penetrate the intestinal mucosa and disseminate via blood and lymphatic circulation to organs where schizogony occurs with the formation of schizonts. These produce micromerozoites that invade leukocytes and differentiate into circulating gamonts, which are infective to ticks during blood feeding. Sexual reproduction subsequently occurs within the vector, leading to oocyst formation and rendering the tick infective to a new vertebrate host after ingestion [2].

Two species are currently recognized as infecting dogs—*Hepatozoon canis* and *Hepatozoon americanum*—which differ in clinical presentation, tissue tropism, pathological findings, and antigenic and genetic characteristics. *Hepatozoon canis* primarily infects leukocytes and is associated with dissemination to organs such as the spleen, liver, and bone marrow, whereas *H. americanum* predominantly forms cysts within skeletal and cardiac muscle tissues, leading to marked myositis [11]. In Brazil, *H. canis* is the only species described. Clinical signs of hepatozoonosis caused by this species are nonspecific and may include anemia, weight loss, and peripheral lymphadenopathy [2].

The diagnosis of these hemoparasites relies on an integrated approach combining clinical findings with laboratory methods, including microscopic, serological, and molecular techniques. Among these, polymerase chain reaction (PCR)-based assays provide the highest diagnostic sensitivity, with amplification of the 18S rRNA gene being the most widely used molecular marker for detection and species characterization [1,2].

Both babesiosis and hepatozoonosis are highly relevant to small-animal clinical practice due to the widespread distribution of *Rhipicephalus sanguineus sensu lato* in Brazil. This tick is the sole vector of *B. vogeli* and the principal vector of *H. canis* in the country [9,11]. Although canine babesiosis is relatively well studied, data on the occurrence of *B. vogeli* in several cities of the metropolitan region of Rio de Janeiro remain limited. Additionally, information on *H. canis* is even scarcer in this state. Therefore, epidemiological investigations employing sensitive and specific molecular diagnostic tools are required to better determine the occurrence of these agents in Rio de Janeiro State. Based on this context, this study aimed to determine the frequency and potential risk factors associated with infection by piroplasmids and *Hepatozoon* spp. in cities in the metropolitan region of Rio de Janeiro, Brazil, using molecular analyses of canine blood samples.

## 2. Materials and Methods

### 2.1. Sampling and Study Areas

Blood sampling and the collection of animal data were conducted through active surveillance. Dog owners were approached and invited to participate in the study on previously scheduled dates, in collaboration with residents’ associations from different Administrative Regions of the study area, as well as through door-to-door visits. All owners who agreed to participate signed an Informed Consent Form prior to sample collection.

A total of 372 canine blood samples were included in this study. These were collected between June and October 2023. Of these, 105 originated from Maricá, designated as Area 1 (A1), and 267 from the Administrative Regions of Barra da Tijuca, Guaratiba, and Jacarepaguá, designated as Area 2 (A2), located in the municipality of Rio de Janeiro.

These areas were selected based on three criteria: ongoing anthropogenic pressure, direct interface with conservation areas, and consequently, their potential for vector and wildlife host circulation.

Convenience sampling was employed, as no previous sample size calculation was performed. Subsequently, sample size estimation was conducted using the StatCalc tool of Epi Info^®^ 7.2.6.0 (CDC), assuming an unknown canine population, to assess the statistical robustness of the obtained sample (*n* = 372).

Due to the scarcity of molecular epidemiological data on the frequency of piroplasmids and *Hepatozoon* spp. in Rio de Janeiro State, the parameters used for sample size estimation were based on previous studies conducted in metropolitan areas with similar epidemiological profiles. An expected prevalence of 39.2% for *Hepatozoon* spp. [12] and 9.3% for piroplasmids [13] was adopted. Based on these assumptions, minimum sample sizes of 157, 258, and 366 dogs would provide confidence levels of 80%, 90%, and 95%, respectively, indicating adequate statistical confidence for the analyzed sample.

The municipality of Maricá, in the state of Rio de Janeiro, has a territorial area of 361.572 km^2^. According to the 2022 Census of the Brazilian Institute of Geography and Statistics [14], the resident population was 197,277 inhabitants. The Municipal Human Development Index in 2010 was 0.765, classified as high [15]. Approximately 98% of the population lived in urban areas [16]. Maricá comprises coastal and urbanized regions, as well as remaining fragments of Atlantic Forest vegetation. It borders the Serra da Tiririca State Park, a conservation area created to protect a coastal mountain range between Maricá and Niterói. In recent years, Maricá has undergone accelerated urban transformation driven by increasing pre-salt oil royalty revenue after 2006, fostering real-estate expansion, occupation of forest-edge areas, and intensification of the urban–wildlife interface [17].

The Administrative Regions of Barra da Tijuca, Guaratiba, and Jacarepaguá (A2) are located in the West Zone of Rio de Janeiro. Barra da Tijuca covers approximately 165.6 km^2^, with an estimated population of 421,438 inhabitants in 2022 and a population density of about 2528 inhabitants/km^2^ [18]. The region has a Human Development Index of 0.918, categorized as high. Jacarepaguá spans 126.61 km^2^ and houses 653,492 inhabitants [19]. Guaratiba covers 139.5 km^2^ and has experienced notable population growth, increasing from 123,114 inhabitants in 2010 to 176,929 in 2022 [20].

These regions contain some of the most important conservation areas in the city, including large forest massifs, restinga ecosystems, mangroves, and coastal lagoons—key environments for the preservation of the Atlantic Forest. Among the most significant areas is the Pedra Branca State Park, which covers portions of Jacarepaguá and Guaratiba [21]. In Barra da Tijuca, the Marapendi Municipal Natural Park and the Bosque da Barra Municipal Natural Park protect restingas, wetlands, lagoons, and remaining coastal vegetation, forming important ecological corridors. Guaratiba also includes the Guaratiba Biological Reserve [22,23].

These Administrative Regions represent some of the most recently populated and urbanized areas of the West Zone, with accelerated growth especially after the 1970s. This expansion was driven by new road construction, real-estate development, and housing policies, resulting in numerous high-end residential complexes and increased density in planned neighborhoods. However, areas surrounding conservation areas face intense pressure from unregulated urban expansion, including the growth of informal settlements and new condominiums in environmentally sensitive zones—a trend that has intensified recent decades [23].

### 2.2. Collection of Biological Samples

Blood samples were collected from adult dogs aged one year or older, regardless of breed, sex, or reproductive status. Between 2 and 5 mL of blood was obtained by cephalic, femoral, or jugular venipuncture while the animals were manually restrained by their pet owner. Sampling was performed using intravenous infusion sets, and samples were immediately transferred to EDTA tubes, properly identified, and stored at 4 °C. Upon arrival at the Parasitology Laboratory of the Universidade Federal Fluminense (UFF), samples were aliquoted into 1.5 mL microtubes and stored at −20 °C until processing.

### 2.3. Molecular Diagnosis of Parasites

All blood samples were subjected to molecular analysis at the UFF Parasitology Laboratory. DNA was extracted using the Roche^®^ kit (Mannheim, Germany), following the manufacturer’s instructions. The extracted DNA was initially submitted to PCR targeting the GAPDH gene, as previously described [24], to confirm the quality of the extraction.

For piroplasm detection, PCR was performed using Promega^®^ Master Mix (Madison, WI, USA) and the primers Bab 143-167 and Bab 858-834 (Thermofiher^®^, São Paulo, Brazil), as previously described [5], which amplify ~700 bp fragments of the 18S rRNA gene from *Babesia*, *Rangelia*, and *Theileria*. The reactions were performed in a thermocycler under the following cycling conditions: initial denaturation at 95 °C for 5 min, followed by 40 cycles of denaturation at 95 °C for 1 min, annealing at 64 °C for 1 min, and extension at 72 °C for 1 min, with a final extension at 72 °C for 5 min.

Detection of *Hepatozoon* spp. followed two independent PCR protocols, both targeting the 18S rRNA gene. The first involved a nested PCR, using primers HAM-1 and HPF-2 in the first reaction [25] and primers 4558 and 2733 in the second reaction [26]. This nested assay yields a 1120 bp product. Primary PCR conditions consisted of an initial denaturation at 95 °C for 3 min, followed by 40 cycles (95 °C for 1 min, 56 °C for 1 min, and 72 °C for 90 s) and a final extension at 72 °C for 7 min. The secondary PCR included an initial denaturation at 94 °C for 3 min, followed by 40 cycles (94 °C for 1 min, 55 °C for 2 min, and 72 °C for 2 min) with a final extension at 72 °C for 10 min.

The second protocol employed conventional PCR using primers HepF300 and Hep900, which amplify a 600 bp fragment, as previously described [27]. The PCR was performed with an initial denaturation at 94 °C for 3 min, followed by 35 cycles of denaturation at 94 °C for 45 s, annealing at 56 °C for 1 min, and extension at 72 °C for 1 min, with a final extension at 72 °C for 7 min.

The reactions for the detection of piroplasmids and *Hepatozoon* spp. were performed using 2.5 µL of each primer (10 pmol), 12.5 µL of Master Mix, 5 µL of DNA, and 2.5 µL of ultrapure water. However, the second round of the nested PCR for *Hepatozoon* spp. was carried out using 1.5 µL of DNA from the first PCR product and 6.5 µL of ultrapure water.

All PCR assays were performed using negative controls consisting of ultrapure water and positive controls obtained from samples generated in parallel projects conducted in our laboratory [13,28], as well as from material kindly provided by collaborating laboratories. All PCR products were purified using ExoSap PCR Product Cleanup Reagent (Applied Biosystems™, Thermo Fisher Scientific, Waltham, MA, USA), following the manufacturer’s recommendations. Sequencing was performed using the same primers employed in the PCR on a 3730xl DNA Analyzer (Applied Biosystems^®^, Carlsbad, CA, USA) with the Fundação Oswaldo Cruz platform. Sequence trimming and consensus assembly were conducted in BioEdit software version 5.0.9 [29]. Consensus sequences were saved in FASTA format and aligned with homologous sequences from GenBank using MEGA-X software (Philadelphia, PA, USA) [30]. Phylogenetic inference was performed using the Maximum Likelihood method with bootstrap support based on 1000 replicates. The best evolutionary model was selected using the Akaike Information Criterion (AIC) in W-IQ-Tree (http://iqtree.cibiv.univie.ac.at/ (accessed on 1 November 2025)). Phylogenetic tree editing and rooting were performed in MEGA-X and iTOL (Heidelberg, Germany) [31] software.

All sequences generated in this study were deposited in the GenBank database (Table S1). The accession numbers PX781445–PX781457 confirmed the molecular identification of the piroplasmid. For *Hepatozoon* spp., sequences generated from different molecular targets and amplification protocols were deposited separately. Sequences obtained using the nested PCR protocol were deposited under accession numbers PX868918–PX868925, whereas sequences amplified by conventional PCR were deposited under accession numbers PX783242–PX783247 (Table S1).

### 2.4. Questionnaires Completed by Pet Owners and Statistical Analysis

The frequency of positive blood samples was determined based on the combined results obtained from three independent PCR protocols, one targeting piroplasmids and two targeting *Hepatozoon* spp. PCR positivity was considered as molecular evidence of hemoparasite DNA in the analyzed samples. Because clinical samples may contain low parasitemia or degraded DNA, not all PCR-positive samples yielded nucleotide sequences of sufficient quality for phylogenetic analysis. Therefore, the epidemiological frequency data and the risk factor analysis for piroplasmids and *Hepatozoon* spp. were estimated based on the molecular evidence generated by PCR, whereas sequencing was performed exclusively to confirm species identity in representative samples.

In addition to blood collection, general information and management data were obtained through questioning pet owners. The retrieved variables included estimated home location, estimated age, breed, sex, predominant coat color, coat length, presence of undercoat, estimated weight, time spent inside or outside the household, whether the dog traveled with its owner, travel destinations, clinical signs such as lethargy and weight loss, previous history of “tick-borne disease,” treatment history, and the drug used if treatment had occurred. All data were stored in Microsoft Excel spreadsheets.

Statistical analyses were performed to assess whether positivity for hemoparasites was significantly associated with the variables collected through the questionnaires. Initially, a univariate screening analysis was conducted using Fisher’s exact test or the Chi-square test, adopting a significance threshold of *p* ≤ 0.2. Variables that were significant in the univariate analysis were subsequently included in a multivariate logistic regression model to evaluate their combined effects. Odds ratios were calculated for each variable included in the model. Variables were considered statistically significant when *p* ≤ 0.05, as previously determined in prior studies [13,28,32]. All analyses were performed using Epi Info software, version 7.2.6.0.

Finally, results referring to positive dog samples were georeferenced and plotted on maps of the study areas to illustrate spatial distribution and occurrence hotspots of the parasites. All maps were created using ArcGIS version 10.

## 3. Results

### 3.1. Frequency of Hemoparasites: Piroplasmids and Hepatozoon *spp.* by Study Area

Molecular screening by PCR, employing different genetic targets, identified 30 positive dog samples among the 372 analyzed (8.1%) for hemoparasites (piroplasmids and *Hepatozoon* spp.). Stratification by sampling area showed that 11/105 (10.5%) of the positive animal samples originated from Area 1, whereas 19/267 (7.1%) were from Area 2, with no statistically significant difference between areas (*p* = 0.29) (Table 1 and Figure S1).

When considering piroplasm infections only, 13/372 dog samples (3.5%) were confirmed positive, with a higher proportion observed in Area 1 (7/105; 6.6%) compared to Area 2 (6/267; 2.2%). This difference was statistically significant (*p* = 0.05), indicating a greater occurrence of piroplasmids in dogs from Area 1 (Table 1).

For *Hepatozoon* spp., Area 2 exhibited the highest frequency of positive dog samples, with 15/267 (5.6%), while Area 1 presented 5 positive dog samples (4.7%). In total, 20/372 dog samples (5.4%) tested positive for *Hepatozoon* spp. No statistically significant difference was observed between the areas for this agent (*p* = 1.00) (Table 1).

**Figure 1:**
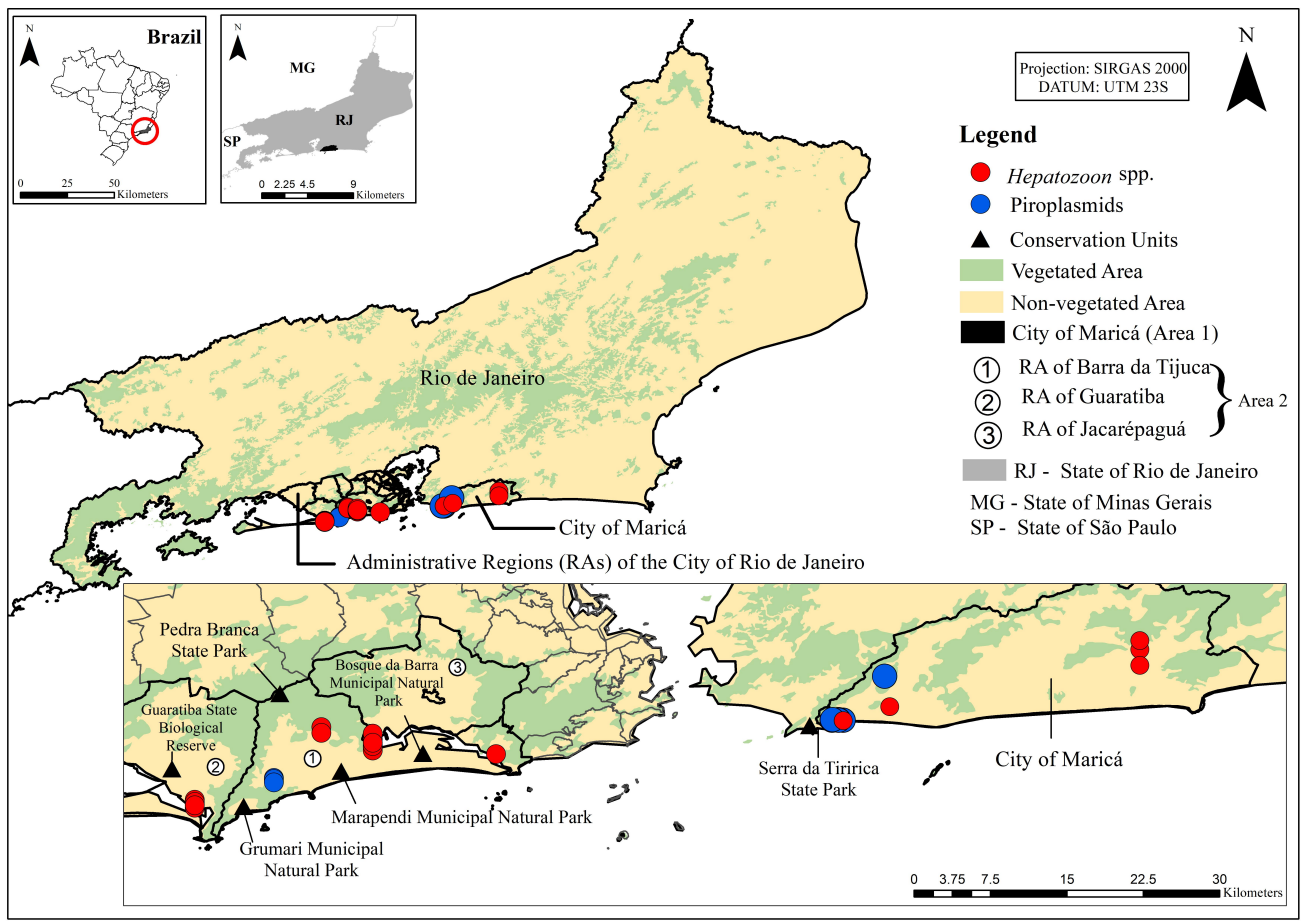
Spatial distribution of positive samples for *Hepatozoon* spp. and piroplasmids in dogs residing in the state of Rio de Janeiro, Brazil. The points indicate detection sites of *Hepatozoon* spp. (red circles) and piroplasmids (blue circles) in the sampled municipalities. Shaded areas represent regions with vegetation cover and non-vegetated areas, as well as the location of conservation units. Maps were generated using the SIRGAS 2000 geodetic reference system, UTM projection, zone 23S.

Positive dog samples for *Hepatozoon* spp. and piroplasmids were recorded in all areas of the state of Rio de Janeiro, including the municipality of Maricá and the Administrative Regions of Barra da Tijuca, Guaratiba, and Jacarepaguá. Detections occurred both in urban zones and in transition areas adjacent to vegetation fragments and conservation areas. No spatial segregation was observed between cases positive for *Hepatozoon* spp. and piroplasmids, nor was any distinct geographic pattern identified among the evaluated areas (Figure 1).

### 3.2. Demographic Characteristics and Risk Factor Analysis Associated with Hemoparasite Infection

The canine population included in the study was predominantly composed of animals up to 8 years of age (282/372; 75.8%) and without a defined breed (mixed breed; 255/372; 68.5%). There was a slight predominance of females (192/372; 51.6%) compared with males (180/372; 48.3%). Regarding phenotypic characteristics, most dogs did not present with a black coat color, being classified under other coat colors (238/372; 63.9%), had short hair (253/372; 68.0%), and lacked an undercoat (322/372; 86.5%).

With respect to household management, most animals predominantly remained indoors (204/372; 54.8%), had been living with the same owner for up to five years (218/372; 58.6%), and did not have a history of travel to other locations (331/372; 89.4%). Regarding clinical manifestations, most owners reported the absence of signs such as lethargy (“tiredness”) (324/372; 87%), weight loss (348/372; 93.5%), and previous history of “tick-borne disease” (334/372; 89.7%) (Table 2).

Overall, when combining results from Areas A1 and A2 and correlating hemoparasite frequency with the evaluated variables, univariate screening analysis showed that age, time living at the current residence, and history of tick-borne disease were significantly associated with infection. Dogs older than eight years had a higher detection frequency (4.8%) than younger animals (3.2%) (*p* = 0.04). Animals that had lived in the same household for more than five years also showed higher prevalence (4.6%) compared with those living there for a shorter period (3.5%) (*p* = 0.08). Moreover, absence of a known history of tick-borne disease showed the strongest association, with 6.2% positivity among dogs without prior clinical history versus 1.9% among those with previous disease (*p* = 0.02). All other variables—including breed, sex, coat color, coat length, presence of undercoat, weight, indoor/outdoor habits, travel history, and owner-reported lethargy or weight loss—showed no statistically significant association (*p* > 0.2).

When these variables were evaluated together in the multivariate logistic regression model, a lack of previous knowledge of tick-borne disease remained significantly associated with infection. Dogs without a known history of the disease were nearly three times more likely to test positive for the hemoparasites studied compared with those with reported prior disease (OR = 2.98; *p* = 0.02) (Table 2).

The evaluation of Areas A1 and A2 revealed distinct patterns in hemoparasite frequency. In Area A1, none of the assessed variables showed an association with infection in the univariate analysis (*p* > 0.2). Detection frequencies remained low and relatively homogeneous across categories, ranging from 0 to 10.5%.

In Area A2, although most variables also showed no significant association in the univariate analysis, two factors demonstrated either a trend or statistical significance (*p* ≤ 0.2). Residence time greater than five years exhibited a marginal association with higher hemoparasite occurrence (4.1% vs. 3.0%; *p* = 0.05), leading to its inclusion in the multivariate analysis. However, after adjustment, this variable did not remain significant (*p* = 0.06; OR = 2.50; 95% CI: 0.94–6.64), suggesting no independent effect. Conversely, the absence of a reported history of tick-borne disease—based on owner information—showed the strongest association in Area A2. Dogs whose owners reported no prior tick-borne disease exhibited higher infection frequency (4.5%) compared with those with such history (2.6%), resulting in a significant association in the univariate analysis (*p* = 0.001). In the multivariate model, this variable remained independently associated with hemoparasite detection, with a marked increase in the odds of positivity (OR = 6.13; 95% CI: 2.1–17.5; *p* = 0.0007) (Table 2).

### 3.3. Detection Patterns and Co-Occurrence of Piroplasmids and Hepatozoon *spp.* in Dogs Based on PCR Assays

When the results obtained for piroplasm detection were integrated with those generated by the two independent PCR protocols targeting *Hepatozoon* spp., most samples were positive for a single pathogen. In total, 13 animals were positive for piroplasmids, of which 10 were exclusively positive for piroplasmids. Co-positivity between piroplasmids and *Hepatozoon* spp. was identified in three dogs. One animal tested positive simultaneously for piroplasmids and *Hepatozoon* spp. by nested PCR, whereas two animals were positive for piroplasmids and concurrently positive in both PCR assays targeting *Hepatozoon* spp.

**Figure 2:**
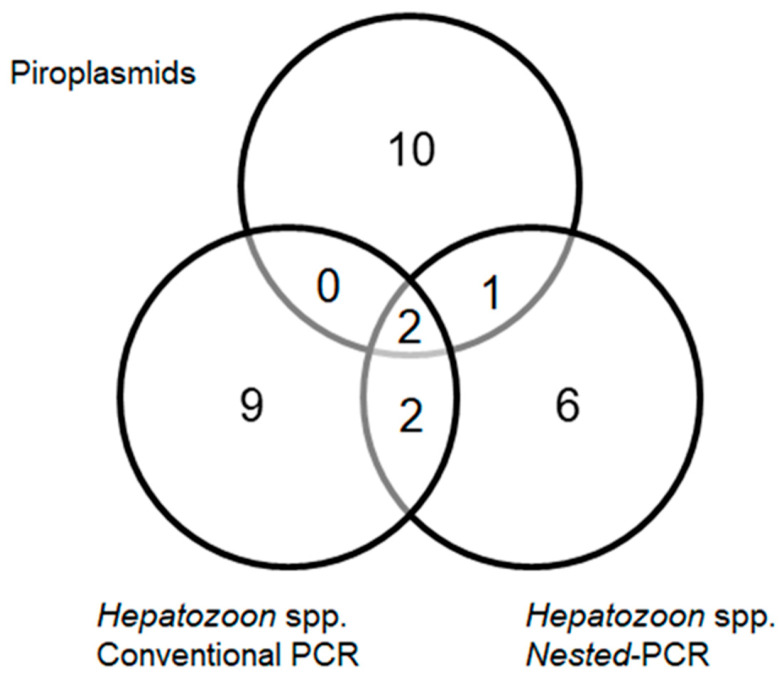
Distribution and overlap of detections of piroplasmids and *Hepatozoon* spp. obtained by nested and conventional PCR, illustrated through a Venn diagram.

Inspection of the Venn diagram summarizing the PCR results for *Hepatozoon* spp. showed that nested PCR detected a total of 11 positive samples, whereas conventional PCR identified 13 positive samples. Notably, four dogs were simultaneously positive for *Hepatozoon* spp. by both conventional and nested PCR, representing the overlapping portion of the Venn diagram. In contrast, nine dogs were detected exclusively by conventional PCR and six exclusively by nested PCR (Figure 2).

### 3.4. Species Confirmation and Phylogenetic Relationships

**Figure 3:**
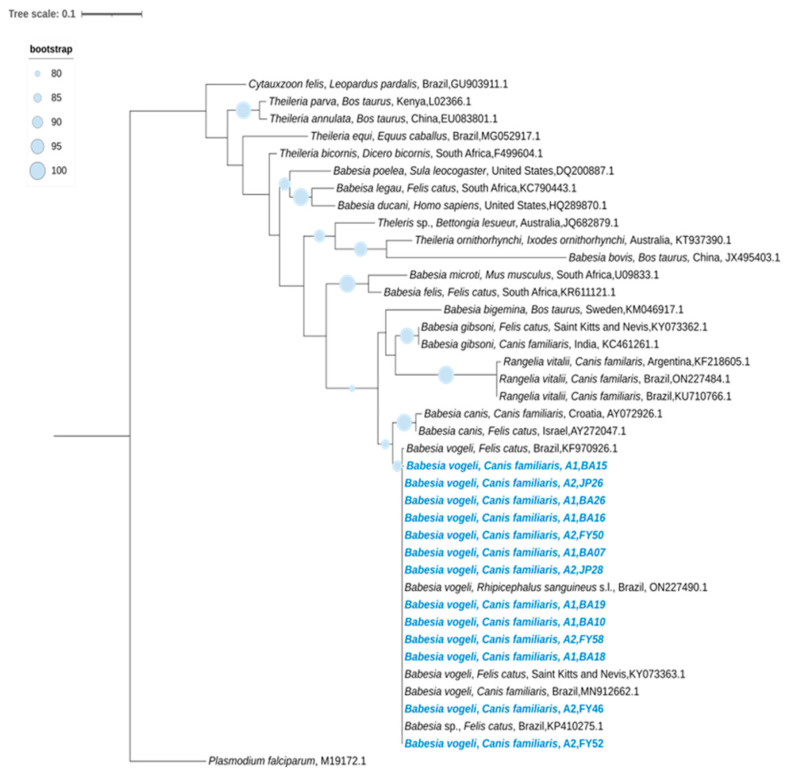
Phylogenetic tree inferred from the alignment of a 676 bp fragment of the 18S rRNA gene of piroplasmids, including *Babesia vogeli* sequences obtained from blood samples of dogs from different areas of the state of Rio de Janeiro, Brazil, highlighted in blue (Area 1: Maricá; Area 2: Administrative Regions of Barra da Tijuca, Guaratiba, and Jacarepaguá). The phylogenetic reconstruction was performed using the Maximum Likelihood method under the TIM2+F+I+G4 evolutionary model. Bootstrap support values, calculated from 1000 replicates, are shown at the nodes. *Plasmodium falciparum* was used as the outgroup for tree rooting.

All 13 blood samples positive for piroplasmids were confirmed by Sanger sequencing to be *Babesia vogeli*. Phylogenetic analysis showed that all sequences clustered in a single well-supported monophyletic clade together with reference sequences of *B. vogeli* previously reported in dogs, domestic cats, and ixodid ticks from Brazil, as well as a domestic cat from Saint Kitts and Nevis. No phylogenetic segregation associated with host species or geographic origin was observed. The sequences obtained in this study exhibited nucleotide identity values ranging from 99.52% to 100%, with 100% query coverage when compared with *Babesia vogeli* reference sequences. Additionally, the sequences showed nucleotide identity ranging from 96.8% to 97.6%, with 100% query coverage, when compared with *Babesia canis* sequences available in GenBank (Figure 3).

**Figure 4:**
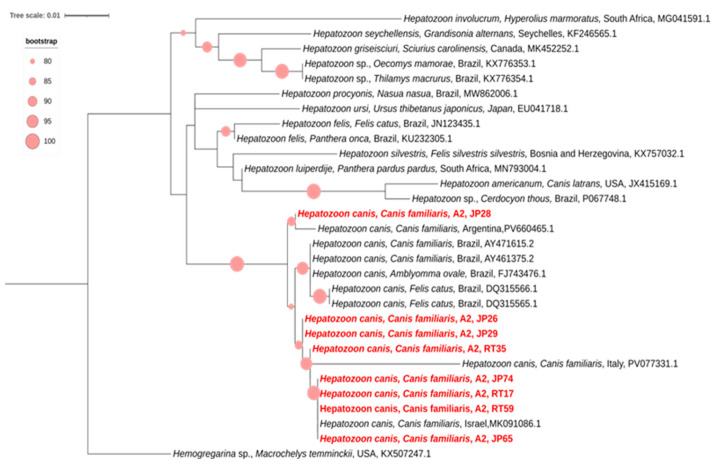
Phylogenetic tree inferred from the alignment of a 624 bp fragment of the 18S rRNA gene of *Hepatozoon* spp., including *Hepatozoon canis* sequences obtained by nested PCR from blood samples from dogs from different areas of the state of Rio de Janeiro, Brazil, highlighted in light red (Area 1: Maricá; Area 2: Administrative Regions of Barra da Tijuca, Guaratiba, and Jacarepaguá). Phylogenetic reconstruction was performed using the Maximum Likelihood method under the K3Pu+F+G4 evolutionary model. Bootstrap support values, calculated from 1000 replicates, are shown at the branches. *Hemogregarina* sp. was used as the outgroup for tree rooting. The bar represents the number of substitutions per site.

Of the 11 samples positive for *Hepatozoon* spp. in the nested PCR, eight generated high-quality nucleotide sequences, all confirmed as *Hepatozoon canis*. These sequences were obtained from blood samples of dogs residing in Area 2. In the phylogenetic analysis, all sequences from the present study clustered within a well-supported monophyletic clade (bootstrap = 100) composed exclusively of reference sequences of *H. canis* (Figure 4).

Within this clade, the sequences obtained in this study showed nucleotide identity values ranging from 97% to 100% when compared with *H. canis* sequences available in GenBank. Sequence JP28 clustered most closely with a reference *H. canis* sequence from a dog in Argentina, sharing 100% nucleotide identity. Sequences JP26, JP29, and RT35 grouped with a reference sequence from a dog in Italy, showing 97% identity. Meanwhile, sequences JP74, JP65, RT17, and RT59 formed a subclade together with an *H. canis* sequence isolated from a dog in Israel, with 100% identity (Figure 4). Overall, the intraspecific nucleotide divergence among the sequences generated in this study ranged from 0% to 3%.

**Figure 5:**
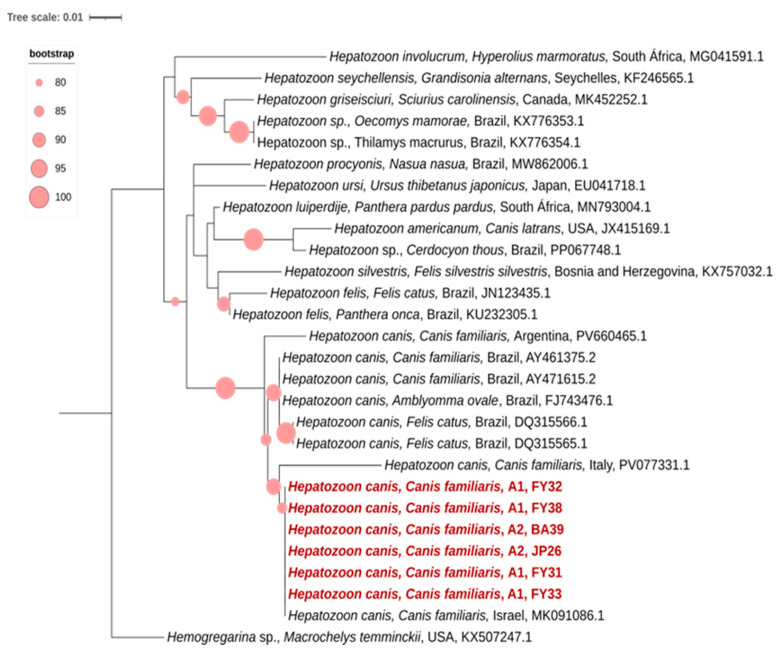
Phylogenetic tree based on the alignment of a 624 bp fragment of the 18S rRNA gene of *Hepatozoon* spp., including *Hepatozoon canis* sequences obtained by conventional PCR from blood samples from domestic dogs from different areas of the state of Rio de Janeiro, highlighted in dark red (Area 1: Maricá; Area 2: Administrative Regions of Barra da Tijuca, Guaratiba, and Jacarepaguá). The phylogenetic analysis was conducted using the Maximum Likelihood method under the K3Pu+F+G4 evolutionary model. Bootstrap values (1000 replicates) are shown at the branches. *Hemogregarina* sp. was used as the outgroup.

Using the conventional PCR protocol, of the 13 samples positive for *Hepatozoon* spp., only six generated nucleotide sequences of sufficient quality for analysis, all confirmed to be *Hepatozoon canis*. In the phylogenetic tree, these sequences clustered into a single, well-supported monophyletic clade (bootstrap = 100), which includes reference sequences of *H. canis* isolated from dogs in different countries. Notably, the *H. canis* sequences obtained in this study showed greater phylogenetic proximity to isolates from Israel than to *H. canis* sequences previously described from dogs in Brazil. Comparison of nucleotide identity between the obtained sequences and those from Argentina and Israel revealed identity values ranging from 98.86% to 100%, respectively (Figure 5).

The distribution of PCR-positive samples, including hemoparasite detection, amplification protocols, and sequencing outcomes, is presented in the flowchart shown in Figure S1, which summarizes the molecular workflow and the origin of positive samples analyzed in this study.

## 4. Discussion

The overall positivity for hemoparasites, piroplasmids, and *Hepatozoon* spp. detected through different PCR protocols indicates the circulation of these agents in the canine population residing in different areas of the state of Rio de Janeiro. This pattern appears to be supported by the endemicity of the ixodid *Rhipicephalus linnaei*, the principal vector, across several regions of the state. It is important to note that the diagnosis of piroplasmids in dogs in Rio de Janeiro has already been reported in multiple studies, including rural, urban, and highland areas [12,13,33,34,35,36,37]. However, reports of *Hepatozoon* spp. infections in dogs from the state are scarce, with only three studies identified: one clinical case report from Campos dos Goytacazes; one epidemiological survey in inland municipalities such as Seropédica, Itaguaí, Paracambi, Mangaratiba, Barra do Piraí, Piraí, and Miguel Pereira, which relied solely on light microscopy; and a third study conducted in the metropolitan region but without specifying the dogs’ area of residence and including only 12 animals [12,38,39].

PCR-based detection of these hemoparasites in both areas does not appear to represent an isolated event in the surveyed locations but rather reflects an epidemiological scenario for ixodid-borne hemoparasites, particularly piroplasmids. The absence of significant differences in the overall frequency of hemoparasites between the canine populations of both areas—considering both piroplasmids and *Hepatozoon* spp.—reinforces the idea that the risk of infection is not restricted to a specific territory. Instead, it suggests a homogeneous exposure to the vector *R. linnaei* across the studied regions.

The molecular evidence of these parasites in the blood of dogs from Areas 1 and 2 may be related to the proximity of residences to conservation areas, notably the Serra da Tiririca State Park near Maricá, the municipal conservation areas adjacent to the Administrative Region of Barra da Tijuca, and the Pedra Branca State Park, which spans the Administrative Regions of Guaratiba and Jacarepaguá. These settings represent key ecological elements for the circulation of ixodid ticks and the pathogens they transmit to domestic dogs living in these areas. It is well established that conservation areas function as permanent ecological reservoirs of ixodid ticks, supported by a high diversity of wild hosts capable of maintaining parasite cycles independently of the urban environment [40]. It is important to emphasize that convenience sampling was employed due to logistical constraints and the difficulty of accessing certain study areas, where active house-to-house recruitment represented a substantial operational effort. Nevertheless, subsequent sample size estimation demonstrated that the analyzed sample achieved adequate statistical power, reaching the minimum sample size required to ensure a 95% confidence level based on expected prevalence parameters. Although this supports the statistical robustness of the findings, the non-probabilistic sampling design may still limit population representativeness. Therefore, future studies employing probabilistic or stratified sampling strategies are recommended to improve representativeness and strengthen epidemiological inferences regarding the circulation of these and other hemoparasites infecting dogs in Rio de Janeiro.

In the specific case of *R. linnaei*, its high ecological plasticity allows it to act as an interface between forest fragments and anthropogenic urban environments in the state of Rio de Janeiro, including regions affected by real-estate pressure and unregulated urban expansion, as observed in Areas 1 and 2. This bidirectional exchange of ixodid-borne pathogens between urban and wild environments has also been reported in other studies in Brazil, which detected *B. vogeli* and *Hepatozoon* spp. in dogs from coastal areas of Ceará; *Hepatozoon* spp. in blood from domestic dogs and *Lycalopex vetulus* in Brasília; and *B. vogeli* and *Rangelia vitalii* in dogs from the mountain region of Rio de Janeiro [28,41,42], as well as in dogs living near fragmented tropical forest areas in Mexico [43].

Beyond geographic drivers influencing the transmission of ixodid-borne pathogens such as piroplasmids and *Hepatozoon* spp., the climatological conditions of Rio de Janeiro State, characterized by a humid tropical climate, likely promote tick survival, metabolic activity, and host-seeking behavior, thereby sustaining vector populations. Climate change has also emerged as a potentially important ecological driver capable of modifying tick distribution and infestation dynamics. Although the magnitude and direction of these effects remain species-dependent and require further empirical validation [44,45,46], climate-based ecological niche models predict an expansion of environmentally suitable areas for *Rhipicephalus sanguineus* across portions of the American continent, including Brazil [47].

Environmental changes tend to reduce the seasonality of infestations, favoring the maintenance of tick populations throughout the year. A similar scenario may be occurring with *R. linnaei* in the state of Rio de Janeiro, especially when associated with inadequate management practices by dog owners, such as irregular or absent use of acaricides. In addition, the lack of environmental control and the free roaming of dogs in areas with high tick infestation may have favored the persistence of the vector and, consequently, the presence and transmission of piroplasmids and *Hepatozoon* spp. in the domestic dogs [33,34,35,36,37,38,39,40,41,42,43,44,45,46,47,48].

When the parasite groups were analyzed separately by area, PCR results showed a higher occurrence of piroplasmids in Area 1, highlighting a distinct spatial pattern that suggests that local factors may be favoring the transmission of these agents. This may include the arrival of newly infected dogs, possibly driven by local governmental initiatives that have promoted human demographic expansion in this region. Conversely, the homogeneous distribution of *Hepatozoon* spp. between areas suggests a more stable circulation pattern, independent of the territorial characteristics evaluated. Considering that *Hepatozoon* spp. infection occurs primarily through ingestion of infected ticks rather than by tick bite, it is plausible that behavioral factors—such as access to outdoor environments and predation or accidental ingestion of vectors—play a more relevant role than broader environmental differences [11,49].

The combined analysis of PCR results and information obtained through questionnaires applied to dog owners showed that age was not associated with hemoparasite positivity, indicating similar exposure across the evaluated age categories. Concordant findings have been reported in studies involving piroplasmids such as *B. vogeli* conducted in Pernambuco, Rio de Janeiro, and Brasília [13,50,51,52], as well as investigations of *Rangelia vitalii* in Rio de Janeiro [28], *H. canis* in dogs from Ceará, Colombia, and Guadeloupe [41,50,51], and *H. canis*, *B. vogeli*, and *R. vitalii* in Uruguay [52].

In contrast, other studies have shown an association between these infections and puppies, both for *B. vogeli* in different Brazilian states—Maranhão, Paraná, Rio de Janeiro, and Ceará [36,41,53,54,55]—and for *B. vogeli* and *H. canis* in dogs from Cuiabá, Brazil [55], as well as reports of *Hepatozoon* spp. exclusively in puppies in Paraíba, Brazil, and Argentina [56,57]. These findings should be interpreted with caution, as the exclusion of puppies limited the evaluation of early-life susceptibility and may have biased epidemiological inferences toward exposure patterns representative mainly of young adult and older dogs.

Breed (mixed-breed versus purebred) and sex were not associated with positivity for the investigated hemoparasites. These findings corroborate previous studies indicating that the risk of canine hemoparasitosis is driven primarily by environmental factors, vector exposure, and management practices rather than genetic or behavioral differences attributable to breed or sex [13,28,36,53,54,58,59,60].

Coat characteristics, including predominant color, hair length, and the presence of undercoats, showed no association with positivity for the investigated hemoparasites. Although such variables are often considered potential risk or protective factors, particularly because they may influence the visualization, detection, and removal of ticks, the results indicate that, within the evaluated context, these characteristics were not sufficient to modify infection risk. In the literature, few studies have incorporated coat traits into risk analyses for hemoparasites such as piroplasmids and *Hepatozoon* spp. Among the studies identified, only one [36], that investigated *B. vogeli* in dogs from Mangaratiba, Seropédica, and Itaguaí (Rio de Janeiro), evaluated hair length and found no association—consistent with the findings of the present study.

Other variables—including body weight, indoor/outdoor behavior, travel history, and clinical signs such as lethargy (“tiredness”) and weight loss—were also not associated with hemoparasite infection. This suggests similar exposure to vectors, predominantly local transmission, and the frequently subclinical nature of these infections, which limits the usefulness of clinical signs as epidemiological screening criteria. Similar results were reported in studies from Pernambuco, Rio de Janeiro, Mato Grosso, and Ceará, where indoor/peridomestic permanence was not associated with *B. vogeli* positivity [36,41,59,60].

In contrast, the absence of a reported history of tick-borne disease, according to dog owners, emerged as the variable most strongly associated with hemoparasite infection, with this effect primarily driven in Area 2. This seemingly paradoxical result suggests that a negative history does not reflect a true lack of exposure to vectors of piroplasmids and *Hepatozoon* spp. but rather prior underdiagnosis, limited clinical perception by owners, or gaps in veterinary follow-up and systematic ectoparasite control. Owner-reported information is inherently subject to recall bias and subjective interpretation, particularly regarding previous clinical episodes and ectoparasite exposure. Mild, nonspecific, or transient clinical signs—such as lethargy, fever, or reduced appetite—may not be recognized as compatible with tick-borne infections and therefore may be underestimated or misclassified. Likewise, tick exposure is frequently underreported, as infestations may go unnoticed, ticks may detach before being observed, or owners may fail to identify immature stages. Such misclassification of both clinical history and vector exposure can distort epidemiological associations and obscure the true infection dynamics. Thus, in Area 2, a negative reported history of tick-borne disease serves as an indirect indicator of actual exposure to vectors, highlighting the limitations of self-report and reinforcing the need for continuous, independent strategies of surveillance, diagnosis, and tick control—even in the absence of previously recognized clinical episodes.

In this context, amplification of an 18S rRNA gene fragment by PCR was a central strategy, as it maximized the detection capacity for hemoparasites associated with infections of nonspecific or subclinical presentation, particularly in poorly characterized epidemiological settings. Conventional parasitological methods, such as examination of blood smears, while useful in acute infections, present limited sensitivity and depend on adequate quantities of circulating parasite forms for microscopic visualization, which may result in underdiagnosis [9].

Molecular techniques overcome these limitations by providing high sensitivity and specificity, allowing the detection of parasitic DNA regardless of morphological integrity or replicative stage. PCR therefore enables identification of both active infections with low parasitemia—often associated with chronic infections—and the persistence of residual parasitic DNA in animals recovering after therapeutic treatment [13]. Thus, PCR positivity should be interpreted as molecular evidence of parasite DNA detection and does not necessarily indicate active infection or current parasitemia. In this context, the use of PCR not only increased diagnostic accuracy but also served as a robust tool for identifying parasite circulation, contributing to more consistent epidemiological interpretations and the development of more effective surveillance and control strategies.

The higher proportion of dogs testing positive exclusively for piroplasmids or only for *Hepatozoon* spp.—whether by nested PCR or conventional PCR—indicates that infections occurred predominantly as single-agent events. Nonetheless, co-positivity for piroplasmids and *Hepatozoon* spp., although detected at low frequency, was observed in samples from dogs BA07, JP26, and JP28, with the latter two originating from Area 2. Although less frequently discussed, co-positivity between piroplasmids such as *B. vogeli* and *Hepatozoon* spp. has been documented in epidemiological studies of dogs from several regions of Brazil—including Rio de Janeiro, Paraíba, Espírito Santo, and Cuiabá—as well as in other South American countries such as Argentina and Uruguay [12,53,56,57,61,62,63]. In most of these studies, pathogen detection was performed using molecular methods, particularly PCR, which is especially relevant for *Hepatozoon* spp., since morphological identification of gamonts in blood smears has markedly limited sensitivity, especially in subclinical or low-parasitemia infections [2].

From a clinical and sanitary perspective, the detection of coinfections involving piroplasmids and *Hepatozoon* spp. is particularly relevant, as concomitant infection may intensify hematological alterations, prolong parasite persistence, and negatively modulate the immune response, contributing to chronic, recurrent, or atypical clinical presentations. Moreover, coinfection may interfere with therapeutic response, requiring more targeted diagnostic and treatment strategies. This scenario applies to both piroplasmids and *Hepatozoon* spp., reinforcing the importance of molecular methods for accurate detection—not only for epidemiological purposes but also for appropriate clinical management of infected dogs, particularly in regions such as Areas 1 and 2, where epidemiological characterization remains limited.

The distribution of diagnostic results for *Hepatozoon* spp. revealed differences between the PCR protocols employed, highlighting the complexity of molecular detection of these hemoparasites. The occurrence of animals positive exclusively by nested PCR or exclusively by conventional PCR indicates that each protocol may capture partially distinct diagnostic scenarios, likely influenced by parasitemia levels and infection dynamics. Nested PCR is commonly regarded as more sensitive and may facilitate the detection of low-parasitemia infections, often associated with subclinical or chronic stages, which can escape detection by conventional PCR [61]. Conversely, positivity restricted to conventional PCR may reflect infections with higher circulating parasitemia. The limited concordance observed between the two assays suggests that reliance on a single molecular strategy may underestimate the circulation of *Hepatozoon* spp., reinforcing the value of complementary diagnostic approaches for epidemiological investigations.

Despite the higher analytical sensitivity generally attributed to nested PCR, the lower number of detections achieved with this protocol in the present study may be explained by methodological and biological factors. Amplification efficiency can be affected by primer–template mismatches, sequence variability in the target region, fluctuations in parasitemia, and the presence of PCR inhibitors in blood-derived DNA. Furthermore, because nested PCR relies on two sequential amplification steps, reduced efficiency in the first round may compromise the final detection. Therefore, the discordance observed between the assays likely reflects differences in assay design and infection dynamics rather than a clear superiority of one protocol over the other. Nevertheless, the absence of validation using independent diagnostic methods represents a limitation, as it prevents determining which protocol more accurately reflects the true infection status. Future studies integrating standardized molecular approaches with microscopic examination and laboratory validation are needed to improve diagnostic accuracy and refine epidemiological interpretations of canine hepatozoonosis.

Analysis of the Sanger sequencing results showed that all 13 blood samples positive for piroplasmids based on 18S rRNA amplification exhibited high nucleotide identity when compared with *B. vogeli* reference sequences deposited in GenBank. These reference sequences include isolates obtained from dogs in the metropolitan region of Rio de Janeiro, from *Rhipicephalus sanguineus* s.l. collected in the mountain region of the state, from domestic cats in São Paulo and Mato Grosso do Sul, and from a dog from Saint Kitts and Nevis [28,55,62,63,64]. This high degree of genetic similarity underscores the conservation of the 18S rRNA marker in *B. vogeli* and the widespread circulation of this parasite.

The overall positivity of 3.5% for *B. vogeli* observed is consistent with national reports, in which this species has been identified as the primary etiological agent of canine babesiosis in Brazil based on molecular methods. Studies conducted in various regions of the country—including the states of Rio de Janeiro, São Paulo, Paraná, Pernambuco, the Federal District, Mato Grosso, Cuiabá, Ceará, Acre, and Pará—have reported frequencies ranging from 2.1% to 15%, demonstrating the widespread distribution and epidemiological relevance of *B. vogeli* in domestic dogs [13,34,35,36,41,42,54,58,59,60,65,66,67,68,69].

Although *B. vogeli* is the predominant species in Brazil, other clinically significant piroplasmids—those capable of inducing acute disease—have also been identified nationally, including *B. gibsoni* in southern Brazil and *R. vitalii* in municipalities of the southern and southeastern regions, including the mountain region of Rio de Janeiro, particularly in dogs living near Atlantic Forest edges [5,28,34,70,71]. These findings highlight the importance of molecular diagnostics for accurate species differentiation and for guiding appropriate therapeutic decisions.

The use of two independent molecular protocols (nested PCR and conventional PCR), followed by Sanger sequencing, enabled confirmation of 14 nucleotide sequences as *H. canis*, based on comparison with reference sequences available in GenBank. These references include isolates obtained from dogs in Brazil, Argentina, Italy, and Israel, as well as from domestic cats and *Amblyomma ovale* in Brazil, reinforcing the taxonomic consistency of the sequences generated [23,72,73,74,75,76]. Molecular evidence confirmed the positivity of *H. canis* in blood samples from 13 dogs, with only one animal (JP26) yielding high-quality sequences from both protocols.

The clustering of sequence JP28 with a 100% identical isolate from Argentina, the grouping of sequences JP26, JP29, and RT35 with an Italian isolate, and the clustering of sequences JP74, JP65, RT17, and RT59 with an isolate from Israel illustrate the high phylogenetic relatedness between *H. canis* lineages detected in Brazil and those described worldwide. This pattern corroborates the broad geographic distribution of this parasite.

Not all PCR-positive samples for *Hepatozoon* spp. from Areas 1 and 2 yielded interpretable nucleotide sequences—an expected finding in molecular diagnostics of hemoparasites. This limitation may stem from both biological and technical factors, particularly the low parasitemia characteristic of *H. canis* infections, especially in asymptomatic or chronic animals, which reduces the availability of target DNA for amplification and compromises sequencing quality. Additionally, nonspecific or mixed amplifications—particularly in conventional PCR—may generate background noise or multiple DNA products, preventing accurate Sanger sequencing, especially in the presence of degraded DNA, PCR inhibitors, or variability in DNA extraction efficiency.

Thus, the discrepancy between PCR-positive samples and those successfully sequenced does not indicate diagnostic failure but rather reflects inherent limitations of direct sequencing from clinical samples, particularly in low-parasitemia infections. Nevertheless, the valid sequences obtained were sufficient to confirm the circulation of *H. canis* in the metropolitan region of Rio de Janeiro, representing a novel epidemiological finding in Brazil.

Although the overall frequency of *Hepatozoon* spp. detected was 5.4%, and the proportion of samples confirmed to be *H. canis* by Sanger sequencing was slightly lower, the findings remain consistent with previously reported data. In general, molecular-based studies have documented wide variation in the occurrence of this hemoparasite, strongly influenced by geographic region, sampling design, and diagnostic approach used, with prevalences ranging from 1.33% to 58.7% across different Brazilian states, including Ceará, Mato Grosso, Mato Grosso do Sul, Paraná, Espírito Santo, and Rio de Janeiro [38,41,66,72,73,74,75].

This heterogeneity is also reflected in molecular epidemiology studies conducted in other countries, such as Italy, where positivity reached 50.6% in dogs, and in Colombia, Guadeloupe, and Uruguay, where frequencies ranged from 2.1% to 8.7% [50,51,52,76]. Collectively, these findings reinforce that canine hepatozoonosis tends to persist as an endemic and frequently underdiagnosed infection, particularly in areas where environmental factors, continuous vector presence, and low-parasitemia infections favor the silent circulation of the parasite.

Although *H. canis* is the only *Hepatozoon* species reported infecting dogs in Brazil and has a broad geographical distribution—being detected in South America, Africa, Asia, southern Europe, and islands in the Pacific and Indian Oceans [49,77]—*Hepatozoon americanum*, traditionally considered endemic to North America [2], has recently been identified as infecting dogs in Uruguay [52]. Importantly, infection by *H. americanum* is associated with acute and debilitating clinical signs, often progressing to a chronic and relapsing course with significant health impacts [11,77]. Detection of this species in a South American country represents a noteworthy and atypical epidemiological finding, reinforcing the need for species-level molecular identification in studies of canine hepatozoonosis to avoid misinterpretation of the distribution, clinical impact, and circulation dynamics of *Hepatozoon* species.

## 5. Conclusions

In summary, this study demonstrates the circulation of ixodid-borne hemoparasites in dogs from different areas of the state of Rio de Janeiro, located in peri-urban environments and at the interface with conservation areas. The detection of *B. vogeli* and *H. canis* through complementary molecular approaches, supported by sequencing and phylogenetic analysis, revealed an epidemiological scenario characterized predominantly by subclinical infections of low parasitemia—thus highly susceptible to underdiagnosis when conventional diagnostic methods are used alone.

Given this scenario, it is essential that veterinarians act not only in clinical diagnosis and case management but also as key agents in health education. Guiding the public on tick control, preventive management of dogs, and early recognition of infection is crucial in reducing the environmental persistence of these parasites. Accordingly, these findings reinforce the need for integrated diagnostic strategies, continuous epidemiological surveillance, and systematic educational actions—measures that are essential in improving clinical and sanitary practices and mitigating the impact of canine hemoparasitoses in urban and peri-urban settings in Rio de Janeiro, Brazil.

## Figures and Tables

**Table 1:** Frequency of hemoparasites, piroplasmids, and *Hepatozoon* spp. detected in blood samples from dogs collected in different areas of the metropolitan region of Rio de Janeiro.

Location	Total	Hemoparasites	Piroplasmids	*Hepatozoon* spp.
Area 1	105	11 (10.5%)	7 (6.6%)	5 (4.7%)
Area 2	267	19 (7.1%)	6 (2.2%)	15 (5.6%)
*p*-value		*p* = 0.29	*p* = 0.05 *	*p* = 1.00

Area 1: Maricá; Area 2: Administrative Regions of Barra da Tijuca/Guaratiba/Jacarepaguá. * Comparisons between areas were performed using Fisher’s exact test (*p* ≤ 0.05).

**Table 2:** Univariate and multivariate analyses of potential risk factors associated with infection by hemoparasites (piroplasmids and *Hepatozoon* spp.) detected in blood samples from dogs collected in different areas of the metropolitan region of Rio de Janeiro.

Category		Total	Parasites		UA	MA		Total A1	Parasites		UA	Total A2	Parasites		UA	MA	
			*n*	%	*p* Value	*p* Value	Odds Ratio		*n*	%	*p* Value		*n*	%	*p* Value	*p* Value	*Odds Ratio*
**Age**	≤8 years	282	18	4.8	0.04 *	0.22	1.88	67	6	5.7	0.52	215	12	4.5	0.06		
	>8 years	90	12	3.2			(0.67–5.25)	38	5	4.8		52	7	2.6			
**Breed**	Mixed breed	255	20	5.4	1			92	10	9.5	1	163	13	4.9	0.62		
	Purebred	117	10	2.7				13	1	1		104	6	2.2			
**Sex**	Male	180	17	4.6	0.44			54	7	6.7	0.52	126	10	3.7	0.64		
	Female	192	13	3.5				51	4	3.8		141	9	3.4			
**Fur color**	Black	134	14	3.8	0.23			42	5	4.8	0.75	92	9	3.4	0.22		
	Others	238	16	4.3				63	6	5.7		175	10	3.7			
**Hair length**	Short	253	18	4.8	0.31			88	9	8.6	1	165	9	3.4	0.22		
	Medium/long	119	12	3.2				17	2	1.9		102	10	3.7			
**Undercoat**	Yes	50	3	0.8	0.78			11	0	0	0.6	39	3	1.1	0.74		
	No	322	27	7.3				94	11	10.5		228	16	6			
**Weight**	≤15 kg	224	17	4.6	0.7			68	6	5.7		156	11	4.1	1		
	>15 kg	148	13	3.5				37	5	4.8	0.51	111	8	3			
**Home environment**	Indoors	204	17	4.6	0.85			23	1	1	0.44	181	16	6	0.13		
	Outdoors	168	13	3.5				82	10	9.5		86	3	1.1			
**Residence time**	Up to 5 years	218	13	3.5	0.08 *	0.61	1.29	48	5	4.8	1	170	8	3	0.05 *	0.06	2.5
	More than 5 years	154	17	4.6			(0.47–3.51)	57	6	5.7		97	11	4.1			(0.94–6.64)
**Travel history**	Yes	41	4	1.1	0.75			1	0	0	1	40	4	1.5	0.5		
	No	331	26	7				104	11	10.5		227	15	5.6			
**Lethargy**	Yes	48	2	0.5				20	0	0	0.11	28	2	0.7	1		
	No	324	28	7.5	0.4			85	11	10.5		239	17	6.4			
**Weight loss**	Yes	24	2	0.5				14	1	1	1	10	1	0.4	0.52		
	No	348	28	7.5	1			91	10	9.5		257	18	6.7			
**History of tick-borne disease**	No	334	23	6.2	0.02 *	0.02 *	2.98	95	11	10.5	0.59	239	12	4.5	0.001 *	0.0007 *	6.13
	Yes	38	7	1.9			(1.17–7.61)	10	0	0		28	7	2.6			(2.1–17.5)

**Statistical Significance:** Univariate analyses (UA) were considered significant at *p* ≤ 0.2, and multivariate analyses (MA) at *p* ≤ 0.05. * *p*-value statistically significant.

## Data Availability

All data supporting the findings of this study are available within the article and its Supplementary Materials.

## Supplementary Materials

The following supporting information can be downloaded at https://www.mdpi.com/article/10.3390/pathogens15040383/s1, Table S1: Individual epidemiological and diagnostic data of dogs and GenBank accession numbers of hemoparasite sequences generated in this study. Figure S1: Workflow and distribution of PCR positivity and sequencing outcomes for piroplasmids and *Hepatozoon* spp. detected in canine blood samples.
