# Supplementary figures and images for: Molecular Epidemiology of Babesia vogeli and Hepatozoon canis in Dogs from Urban and Peri-Urban Areas of Rio de Janeiro, Brazil
Source: Pathogens. 2026 Apr 2;15(4):383. doi: 10.3390/pathogens15040383 (PMC13118792; doi:10.3390/pathogens15040383)

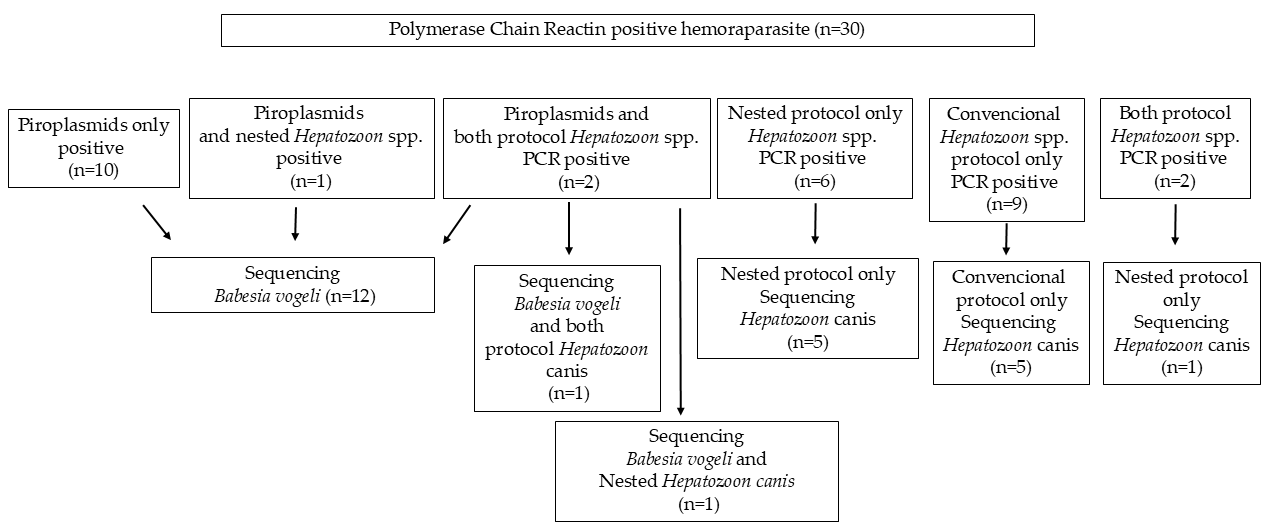

Supplement: Supplementary file 1 [file pathogens-15-00383-s001.zip › Figure S1.tif]
